# Rapid Adsorption of Copper(II) and Lead(II) by Rice Straw/Fe_3_O_4_ Nanocomposite: Optimization, Equilibrium Isotherms, and Adsorption Kinetics Study

**DOI:** 10.1371/journal.pone.0120264

**Published:** 2015-03-27

**Authors:** Roshanak Khandanlou, Mansor B. Ahmad, Hamid Reza Fard Masoumi, Kamyar Shameli, Mahiran Basri, Katayoon Kalantari

**Affiliations:** 1 Department of Chemistry, Faculty of Science, Universiti Putra Malaysia, 43400, Serdang, Selangor, Malaysia; 2 Malaysia-Japan International Institute of Technology (MJIIT), Universiti Teknologi Malaysia, 54100, Kuala Lumpur, Malaysia; Institute for Materials Science, GERMANY

## Abstract

Rice straw/magnetic nanocomposites (RS/Fe_3_O_4_-NCs) were prepared via co-precipitation method for removal of Pb(II) and Cu(II) from aqueous solutions. Response surface methodology (RSM) was utilized to find the optimum conditions for removal of ions. The effects of three independent variables including initial ion concentration, removal time, and adsorbent dosage were investigated on the maximum adsorption of Pb (II) and Cu (II). The optimum conditions for the adsorption of Pb(II) and Cu(II) were obtained (100 and 60 mg/L) of initial ion concentration, (41.96 and 59.35 s) of removal time and 0.13 g of adsorbent for both ions, respectively. The maximum removal efficiencies of Pb(II) and Cu(II) were obtained 96.25% and 75.54%, respectively. In the equilibrium isotherm study, the adsorption data fitted well with the Langmuir isotherm model. The adsorption kinetics was best depicted by the pseudo-second order model. Desorption experiments showed adsorbent can be reused successfully for three adsorption-desorption cycles.

## Introduction

The industrial activities signify an important pollutant source these days, mainly regarding the addition of heavy metals in the soil. This is contributing to a remarkable increase on the concentrations of those ions in waters which represent an important source of contamination of the aquatic bodies, especially when it is considered that this kind of heavy metals be disseminated via the food chain [1].

Heavy metals released into the environment from metal finishing, welding and alloy manufacturing, plating plants and mining cause an important hazard to the environment and public health. The main concern with heavy metals is their ability to accumulate in the environment and leads to heavy metal poisoning. Unlike some organic pollutants, heavy metals are not biodegradable and cannot be metabolized as well as decomposed.

Heavy metals can simply enter the food chain through a number of ways and cause progressive poisonous effects with gradual accumulation in living organisms over their life span [2]. Therefore, it is necessary to control the concentrations of toxic heavy metals in the aquatic environments.

Several treatment techniques have been investigated for the removal of metals from water/wastewater such as membrane filtration, reduction, adsorption/biosorption, ion-exchange, coagulation—flocculation, chemical precipitation, flotation and electrochemical method. Most of them are significantly costly and incapable of removing trace levels of heavy metal ions. Comparatively, adsorption as an exception is the most effective and widely used technique due to higher output and lower cost of absorbents [3]. Therefore, widely available adsorbents with high adsorption capacity should be developed to treat wastewater from toxic heavy metals.

Biosorption is one of the most effective and cost-efficient approaches for removal of heavy metal. Biosorption is a fast and reversible reaction of the heavy metals with biomass. A wide variety of active and inactive organisms have been employed as biosorbent to sequester heavy metal ions from water/wastewater. It has been found that biosorbents are rich in organic ligands or the functional groups, which play a dominant role in the removal of various heavy metal contaminants [4,5]. These biosorbents typically include algae, fungi, rice and wheat straw, hyacinth, pine bark, tea waste, starch, agricultural by-products and microbes [5]. For example some researchers studied the effect of sawdust [6], rice husk [7] and pomegranate peel [8] for removal of copper (II) and lead (II) from aqueous solution.

Rice straw consists of large amounts of cellulose, hemicelluloses and lignin. These compounds can provide binding sites for heavy metals [9]. Neat rice straw has been used in biosorption of heavy metals from aqueous solution, but the yield of heavy metals removal is low and separation of adsorbent from solution is difficult. Coating the Fe_3_O_4_ nanoparticles (Fe_3_O_4_-NPs) on the surface of rice straw causes the yield of heavy metals removal is higher than neat rice straw because of the high surface area to volume ratio of rice straw/Fe_3_O_4_-nanocomposites.

With the rapid development of nanotechnology, it has become possible to fabricate, characterize and specially tailor the functional properties of nanoparticles for different applications. The fabrication of synthetic materials always tries to learn from nature and compete with it in terms of quality and quantity [10]. The widespread application of metal nanoparticles in various areas such as telecommunications and biotechnology has posed new challenges for the controlled synthesis of metal nanoparticles [11].

Increased investigations with several types of iron oxides have been carried out in the field of nanosized magnetic particles, among which Fe_3_O_4_ is a very promising candidate since its biocompatibility has already been proven. With proper surface coatings, these magnetic nanoparticles can be dispersed into suitable solvents, forming homogeneous suspensions [12]. For example Savina et al [13] prepared nanocomposite materials where iron nanoparticles are embedded into the walls of a macroporous polymer and studied their efficiency for the removal of As(III) from aqueous media. Donia et al [14] prepared Nano-magnetic cellulose hybrid for adsorption of some metal ions. Chang et al [15] prepared Fe_3_O_4_/graphene nanocomposites by solvothermal method for adsorption of aniline and *p*-chloroaniline from aqueous solution. Bhaumik et al [16], prepared polypyrrole/Fe_3_O_4_ magnetic nanocomposites for removal of Cr(VI) from aqueous solution and Shen et al [17], studied the application of Fe_3_O_4_ nanoparticles for treating the wastewater contaminated with the metal ions, such as Ni(II), Cu(II), Cd(II) and Cr(VI).

Various adsorption media have been employed for removal of toxic heavy metals. These include biomaterials, metal oxides, hydrous metal oxides and hybrid materials, etc. Some studies have been demonstrated that iron oxide nanoparticles are effective for the transformation of a wide area of common environmental contaminated such as heavy metal ions, chlorinated organic solvents, and various inorganic compounds [18]. Some studies focuses on the heavy metal removal using nanocomposites,

Among heavy metals, lead (Pb) widely exists in soil, air, and water. Lead is a non-biodegradable and easily absorbed and accumulated in living organisms, causing different diseases and disorders such as carcinogenic, genotoxic, anemia, reproductive, and neurological effects, especially to children. Since lead is widely used in mining and metallurgical engineering, battery manufacturing processes and traditional gasoline, the method to separate it from the environment should be seriously studied. Researchers and scientists around the world have indeed paid attention in solving the heavy metal pollution in environment recently [19].

Copper ion displays excessive acute and high chronic toxicity to aquatic organisms that results in the death of the organisms. On the other hand, metals like copper and lead play important roles in most industries. Copper metal has many commercial applications consist of production which is used in engine moving parts, brake linings, copper tubing, wire, metal plating, fungicides, insecticides, copper compounds, brass, bronze and etc. Corrosion of copper metal by water results in conversion of the metal to its ionic forms, Cu^1+^ and Cu^2+^ [20].

This study demonstrates the experimental method for the removal of lead and copper ions from aqueous solutions using response surface methodology (RSM). RSM is a collection of mathematical and statistical techniques useful for analyzing the effect of several factors at different levels and their influence on each other. RSM has an important application in the process design and optimization as well as the improvement of existing design. The aim of RSM would be to optimize the response based on the factors investigated [21]. This methodology is more practical compared to the approaches mentioned above as it arises from experimental methodology which includes interactive effects among the variables and, eventually, it depicts the overall effects of the parameters on the process [22]. It has been successfully applied to optimize in several fields for example in synthesis [23], nanoemulsion formulation [24] and dye removal [18].

In this work the influence of initial ion concentration, removal time and adsorbent dosage on biosorption of Pb(II) and Cu(II) by RS/Fe_3_O_4_-NCs were investigated. The main goal of this work was to find the optimal conditions for removal of lead and copper ions from aqueous solution using RS/Fe_3_O_4_-NCs.

## Materials and Methods

### Materials

All chemical reagents used in this study were of analytical grade and utilized with no further purification. Rice straw was obtained from a local farm (Bukit Tinggi, Kedah, Malaysia). Reagent that were utilized for the preparation of Fe_3_O_4_ nanoparticles are listed as follow: FeCl_3_.6H_2_O and FeCl_2_.4H_2_O (99.89%) were prepared by Merck (Frankfurt, Germany); urea (99%) was obtained from Hamburg Chemicals (Hamburg, Germany); NaOH (99.0%) was supplied by R & M Chemistry (Chicago, USA); and HNO_3_ (70%) and HCl (37%) were obtained from Sigma-Aldrich (St Louis, MO, USA). All solutions were freshly obtained by utilizing double distilled water and put in the dark place to protect any photo-chemical reactions. All glassware utilized in the experimental process were cleaned in a fresh solution of HNO_3_/HCl (3:1, v/v), washed completely with double distilled water, and dried before use.

### Synthesis of rice straw/Fe_3_O_4_ nanocomposites

For the synthesis of RS/Fe_3_O_4_-NCs (20% w/w), constant amount of RS was suspended in deionized water, and then urea solution (2.0 M) was added into the mixture as a stabilizer agent. The FeCl_3_.6H_2_O and FeCl_2_.4H_2_O (2:1 molar ratio) were added into the modified RS suspension with vigorous stirring under nitrogen gas to prevent oxidation of Fe^+2^ in the mixture. Then a freshly prepared NaOH (2.0 M) solution was added into the mixture with a molar ratio of 1:4 under continuous stirring until a black suspension was formed. The suspension was finally centrifuged, washed twice with ethanol and deionized water and dried in oven at 60°C. All the experiments were conducted at ambient temperature [25].

### Characterization methods and instruments

Transmission electron microscopy (TEM) was applied to observe the morphology and measure the size of synthesized samples. A drop of diluted samples in distilled water was trickled on the surface of covered copper grid. TEM images were conducted using a Hitachi H-7100 electron microscope; Powder X-ray diffraction (PXRD) with Cu Kα radiation, PANanalytical, Almelo, the Netherlands, was utilized to determine the samples crystallinity. The surface area of the RS and RS/Fe_3_O_4_-NCs was calculated by a Brunauer, Emmett and Teller (BET) surface area analyzer (JW-04, Beijing). Analyzing the residual ion concentration was carried out by atomic absorption spectroscopy (AAS), thermo Scientific, S series.

### Adsorbate

Two stock solutions (1000 mg/L) of Cu(II), and Pb(II) ions were prepared by dissolving appropriate amounts of copper chloride, and lead nitrate in deionized water and then transfer to the 1-liter volumetric flasks and diluted with deionized water. The stock solutions were then diluted with deionized water to obtain the desired concentration range of Cu(II), and Pb(II) standard solutions.

### Lead and copper ions biosorption studies

Twenty batch biosorption experiments from RSM design were performed at the room temperature to investigate the influence of three independent variables of initial ion concentration, removal time and adsorbent dosage on the ion removal. 25 ml of lead or copper ion solutions with different concentrations poured into Erlenmeyer flask and various amounts of adsorbent were added to the solutions, and stirred with mechanical stirrer in the adjusted agitation speed of 200 r.p.m. The samples were finally filtered and analyzed by atomic absorption spectroscopy (AAS) for determining of residual ion concentration. Metal ion removal by RS/Fe_3_O_4_-NCs was calculated according to Equation (1):

$$P=\frac{C_{0}-\;C_{e}}{C_{0}}\;\times 100$$ (1)

where *P* is the percentage of lead/copper ion adsorbed by biosorbent in percentage, *C*_*0*_ is the initial lead/copper ion concentration in mg/L and *C*_*e*_ is the final lead/copper ion concentration in mg/L.

### Desorption Experiment

To investigate the reusability of the adsorbent, desorption and regeneration of metal-loaded RS/Fe_3_O_4_-NCs were also carried out. After adsorption process the adsorbent was separated magnetically and washed several times with deionized water to remove any unadsorbed metal ions and then desorption studies were performed by mixing resultant metal-loaded adsorbent with 25 mL of 0.10 M HNO_3_ in water shaker bath for 1h. Then the adsorbent was separated magnetically. The drying adsorbent was added to the solution of heavy metals and after stirring with mechanical stirrer was separated from the solution. Then metal-loaded adsorbent was mixed with 25 mL of 0.10 M HNO_3_, shacked and separated from the solution. This process was repeated three times.

### Experimental design

A central composite rotatable design (CCRD) was used to determine the optimum conditions for Pb(II) and Cu(II) biosorption. The optimization studies were conducted by investigation of three independent variables (*k*) consisted of initial ion concentration, removal time and adsorbent dosage. Three independent factors at five levels: factorial points levels (+1 and −1), the replication points level (0), were located in center of cubic design, and the axial points were set at the outer values corresponding to α values (±1. 68) for each of the factors. The Independent variables were coded according to Equation (2):

$$x_{j}=\frac{{\tilde{X}}_{j}-{\tilde{X}}_{j0}}{i_{j}}$$ (2)

where *x*_*j*_ is the variable coded values, ${\tilde{X}}_{j}$ is the variable actual values, ${\tilde{X}}_{j0}$ is the actual value in the center level (coded level, 0), *i*_*j*_ is the variation interval and *j* = 1,2,3 (number of the variable).

A total of 20 experiments were carried out for biosorption optimization. Central points were used to check the reproducibility and stability of results. The runs were performed in a randomized manner to protect against systematic bias. The generalized response surface model is shown by Equation (3), and the variables and their levels chosen for this research were illustrated in Table 1:

$$Y=\beta_{0}+\sum_{i=1}^{2}\beta_{i}x_{i}+\sum_{i=1}^{2}\beta_{ii}x_{i}^{2}+\sum_{i=1}^{2}\sum_{j=2}^{3}\beta_{ij}x_{i}x_{j}+\varepsilon$$ (3)

where Y is the response variable, β_0_ is the constant term, β_i_ is the coefficients of the linear parameters, *χ*_*i*_ shows the variables, β_ii_ demonstrates the coefficients of the quadratic parameter, β_ij_ shows the coefficients of the interaction parameters, and ε is the residual related to the experiments.

**Table 1:** Coded independent variables used in RSM design.

Symbol	Independent variable	Coded levels				
		-α	-1	0	1	α
*X*_1_	Initial ion concentration (mg/l)	32.73	60	100	140	167.27
*X*_2_	Removal time (s)	9.54	30	60	90	110.45
*X*_3_	RS/Fe_3_O_4_-NCs dosage (g)	0.049	0.07	0.1	0.13	0.15

The design of experiment was performed through the Design of Expert (version 7.0.0, StatEase, USA). The design variables which are coded and actual values are calculated by the software based on the values of k and α. Table 2 summarizes the values of each point used for the experimental design of Pb(II) and Cu(II) biosorption.

**Table 2:** Predicted and experimental values of removal of Cu(II) and Pb(II), based on central composite rotatable design (CCRD).

Run	Initial ion concentration (mg/l)	Removal time (s)	Adsorbent amount (g)	Removal of Cu (%)		Removal of Pb (%)	
				Exp.	Pre.	Exp.	Pre.
1	100	60	0.05	60.25	60.65	80.70	80.08
2	60	30	0.07	47.28	47.69	68.18	67.79
3	140	90	0.07	67.89	66.04	84.62	84.92
4	140	30	0.07	69.53	68.78	81.00	81.25
5	60	90	0.07	53.85	54.89	91.18	90.94
6	32.73	60	0.10	74.45	73.24	90.91	91.20
7	100	60	0.10	76.47	77.15	85.02	81.24
8	100	60	0.10	53.35	54.52	76.08	75.68
9	100	60	0.10	73.78	68.78	85.02	81.25
10	100	60	0.10	70.70	68.78	79.18	81.25
11	100	60	0.10	62.21	61.23	73.12	74.62
12	100	9.55	0.10	75.98	77.25	69.41	69.99
13	100	60	0.10	74.50	74.15	89.06	88.22
14	167.27	60	0.10	49.83	50.46	70.24	70.83
15	100	110.45	0.10	67.30	67.43	67.70	65.88
16	140	30	0.13	70.70	68.78	77.72	81.25
17	60	30	0.13	44.37	42.75	59.98	61.10
18	140	90	0.13	46.27	46.68	76.60	75.75
19	60	90	0.13	65.81	68.78	78.28	81.25
20	100	60	0.15	62.31	68.78	86.06	81.25

## Results and Discussion

### Characterization of biosorbent

**Fig 1:**
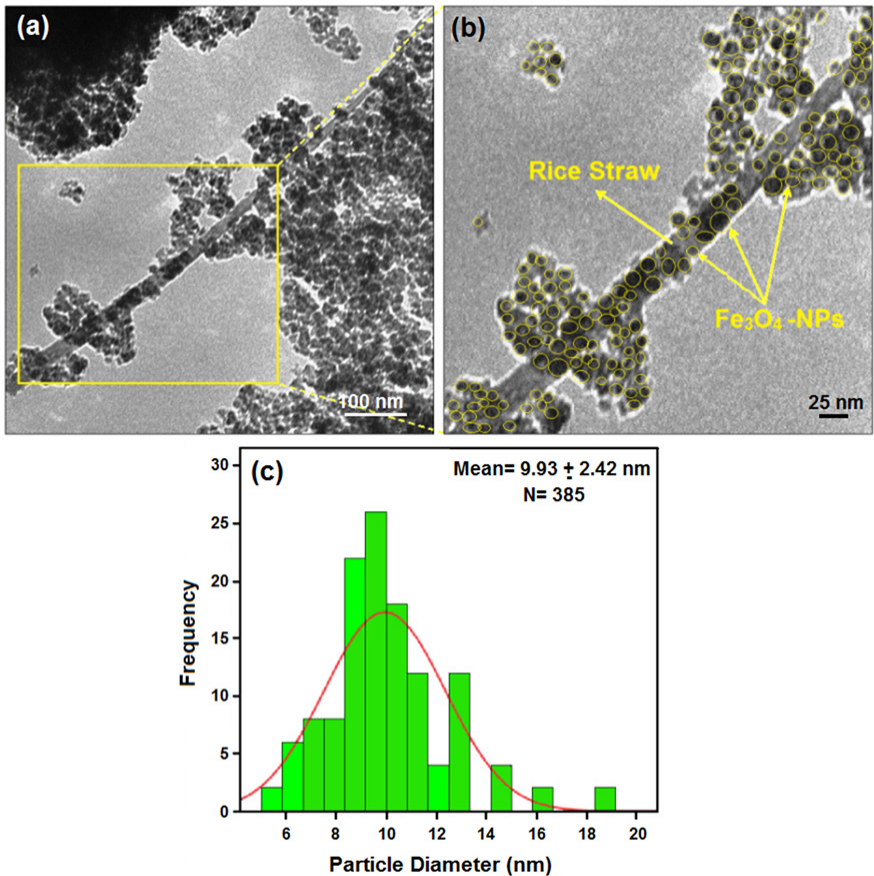
Transmission electron microscopy images and histogram of particle size distribution for rice straw/Fe_3_O_4_-NCs.

Fig. 1 shows transmission electron microscopy (TEM) image and their size distributions. The image indicated which the mean diameters and standard deviation of Fe_3_O_4_ nanoparticles were about 9.93 ± 2.42 nm. Moreover, this confirms the homogeneous dispersion of the Fe_3_O_4_ nanoparticles on the RS surface, even though nanoparticles appear to aggregate to some extent. The Fe_3_O_4_ nanoparticles indicate spherical morphology.

**Fig 2:**
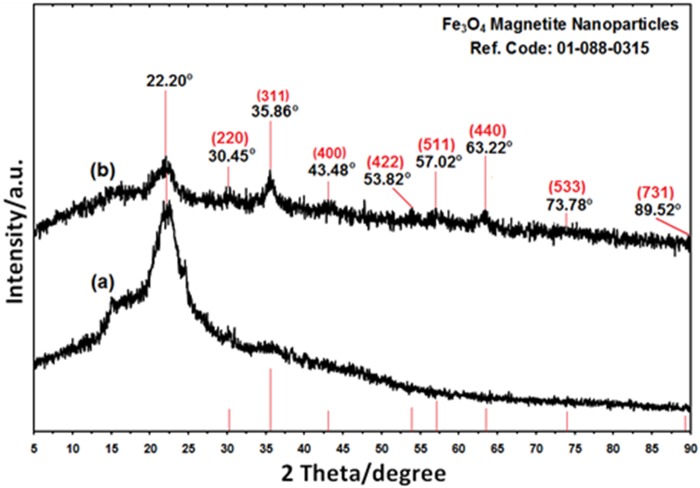
The PXRD of rice straw and rice straw/Fe_3_O_4_-NCs with the related peaks respectively (a,b).

The comparison between the PXRD patterns of the RS and RS/Fe_3_O_4_-NCs in the small angle range of 2θ = 15–25° (Fig. 2) indicated the formation of the intercalated Fe_3_O_4_ nanostructure. In addition to the broad diffraction peak, which was centered at 22.20° is attributed to RS [26,27], eight crystalline peaks were observed at 2θ° of 30.45°, 35.86°, 43.48°, 53.82°, 57.02°, 63.22°, 73.78° and 89.52° related to the 220, 311, 400, 422, 511, 440, 533 and 731 crystallographic planes of face-centered cubic (fcc) iron oxide nanocrystals, respectively (Ref. Code Fe_3_O_4_: 01–088–0315) [25].

### Modeling and analysis of variance (ANOVA)

The statistical significance of the quadratic model was assessed using the analysis of variance (ANOVA) as depicted in Table 3. The fit of the model was evaluated by determination of regression coefficient (R^2^). As indicated in Table 3 the coefficient is 0.9550, 0.9542 for Cu (II) and Pb(II), respectively. The values of R^2^ represent that only 4.5 and 4.58% total variables for Cu(II) and Pb(II), respectively, were not explained by the model. Therefore, the R^2^ values in these regression models are relatively high, which indicate good agreement between predicted and experimental results. The adjusted R^2^ values for Cu(II) and Pb(II) are 0.9051, and 0.9130, respectively. The values of R^2^ and adjusted R^2^ are close to 1 that is extremely high and has advocated a high correlation between the predicted and observed values. The value of predicted R^2^ for Cu(II) is 0.8536 and Pb(II) is 0.8940. The higher values of predicted R^2^ also support a model is highly significant. This also showed that predicted R^2^ values for Cu(II) and Pb(II) are in an appropriate agreement with the adjusted R^2^ values of Cu(II) and Pb(II).

**Table 3:** Analysis of variance of the fitted quadratic equation for removal of Cu(II) and Pb(II), by nano-adsorbent and regression coefficients of the final reduced models.

Source	Removal of Cu (II)					Removal of Pb (II)				
	Sum of squares	DF*	Mean square	*F*-value	*P*-valueProb>*F*	Sum of squares	DF*	Mean square	*F*-value	*P*-value Prob>*F*
Model	2074.06	10	207.41	19.11	<0.0001	1525.09	9	169.45	23.17	< 0.0001
A-Initial ion concentration	114.08	1	114.08	10.51	0.0101	1064.51	1	1064.51	145.53	< 0.0001
B-Time	0.51	1	0.51	0.047	0.8332	76.49	1	76.49	10.46	0.0090
C-Adsorbent dosage	465.35	1	465.35	42.89	0.0001	189.78	1	189.78	25.95	0.0005
AB	2.70	1	2.70	0.25	0.6297	11.96	1	11.96	1.63	0.2300
AC	240.13	1	240.13	22.13	0.0011	2.49	1	2.49	0.34	0.5728
BC	281.68	1	281.68	25.96	0.0006	13.00	1	13.00	1.78	0.2120
A^2^	22.22	1	22.22	2.05	0.1862	0.48	1	0.48	0.066	0.8024
B^2^	840.30	1	840.30	77.44	<0.0001	161.82	1	161.82	22.12	0.0008
C^2^	35.64	1	35.64	3.28	0.1034	0.89	1	0.89	0.12	0.7347
A^2^B	18.43	1	18.43	1.70	0.2248	-	-	-	-	-
Residual	97.65	9	10.85	-	-	73.15	10	7.31	-	-
Lack of fit	14.04	4	3.51	0.21	0.9222	10.18	5	2.04	0.16	0.9665
Pure error	83.61	5	16.72	-	-	62.97	5	12.59	-	-

	**Removal of Cu (II)**	**Removal of Pb (II)**
Standard deviation	3.29	2.70
PRESS	317.97	169.38
R^2^	0.9550	0.9542
Adjusted R^2^	0.9051	0.9130
Predicted R^2^	0.8536	0.8940
CV**	5.20	3.42
Adequate Precision	14.122	18.029

*DF: Degree of Freedom;

**CV: Coefficient of Variation

The significance of the model is confirmed by the smallest value of Prob>F or less than 0.05. The *P*-value shows the interaction strength between each independent variable. As shown in the table the linear term of adsorbent dosage had the most significant effect on the removal of Cu(II) *F*-value (42.89). However the initial ion concentration was found to have the significant effect on the removal of Cu(II) with *F*-value of 10.51. But the removal time didn't show any effect on the Cu(II) removal. On the other hand, the initial ion concentration had the most significant effect on the removal of Pb(II) with a higher *F*-value 145.53. However the adsorbent dosage and removal time were found to have the significant effect on the removal of Pb(II) with *F*-values of 25.95 and 10.46, respectively. For both ions the square of removal time had the significant effect on the removal of Cu(II) and Pb(II) with *F*-values of 77.44 and 22.12, respectively. When the lack of fit values for whole models is non-significant, it displays that the quadratic models were valid [28]. Table 3 shows the *P*-value of the models for both Cu(II) and Pb(II) removal ions is less than 0.05, which displays that the model is regarded being statistically significant. These results revealed that the quadratic model is statistically significant for the response and hence it was used for subsequent analysis. Regression models supply beneficial description of the connection between the independent variables and the response.

In analysis of variance coefficient of variation (CV) for Cu(II) and Pb(II) is 5.20 and 3.42, respectively. The lower values of CV show a high reliability and precision of the experiments [22]. The CV is a measure of reproducibility of the model, moreover as a common rule a model could be considered as reasonably reproducible when its CV is not higher than 10% [29].

The empirical relationship between the removal of Cu (II) and Pb (II) ions (*Y*_1_, *Y*_2_), respectively, and the three variables in coded units obtained by models are shown in Equation (4, 5):

$$Y_{1}=68.78-2.89A-0.30B+5.84C+0.58AB+5.48AC-5.93AC+1.24A^{2}-7.64B^{2}-1.57C^{2}-2.36A^{2}B$$ (4)

$$Y_{2}=81.25-8.83A+2.37B+3.73C+1.22AB-0.56AC-1.27BC-0.18A^{2}-3.35B^{2}+0.25C^{2}$$ (5)

where *Y*_*1*_ and *Y*_*2*_ are removal of Cu(II) and Pb(II) ions in percentage, A, B and C are the coded values of the variables, initial ion concentration in mg/L (A), removal time in second (B) and adsorbent dosage (C) in gram.

**Fig 3:**
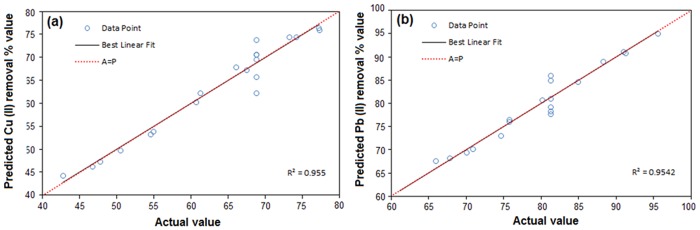
Scatter plot of predicted values versus actual values for (a) Cu(II), and (b) Pb(II).

Fig. 3 summarizes correlation between experimental and predicted values using the developed model. Fig. 3a and b exhibit the actual values versus predicted values of the product, which indicated a good agreement between actual and predicted response.

### Reciprocal effect of variables on the removal of Cu(II) and Pb(II) ions

#### Effect of initial concentration of Cu(II) and Pb(II) and adsorbent dosage on the removal of Cu (II) and Pb (II) ions

**Fig 4:**
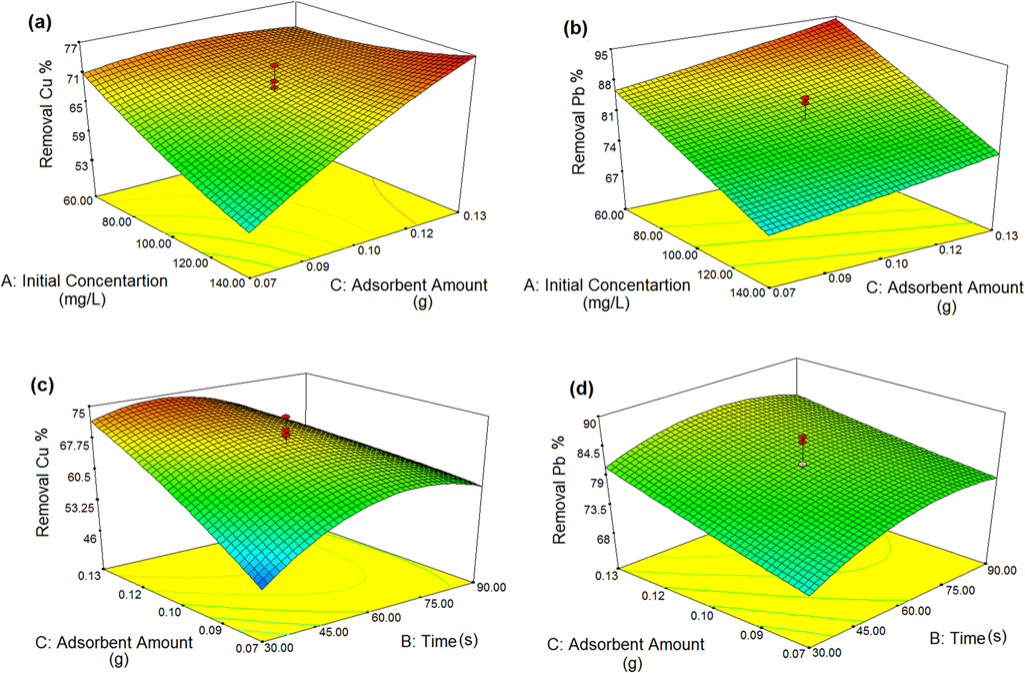
Response surface, showing the effect of initial ion concentration, removal time, and adsorbent dosage on the Cu(II) and Pb(II) percentage removal.

Fig. 4(a, b) indicates the effect of two variables on the removal of Cu(II) and Pb(II) ions from aqueous solutions. The results indicated that initial ion concentration and adsorbent dosage have a strong effect on the sorbent capacity. As can be seen with increasing the initial ion concentration the percentage removal of Cu(II) and Pb(II) was decreased, for example, when Cu(II) and Pb(II) concentrations enhanced from 80 mg/L to 140 mg/L, overall Cu(II) and Pb(II) percentage removal decreased from 70% to 45%, and 77% to 59%, respectively. Maximum Cu(II) and Pb(II) removal percentage was obtained when there is increasing in adsorbent dosage. By increasing of adsorbent dosage, the removal efficiency of ion was increased due to that activated surface of adsorbent possess more adsorption sites available for ions removal from the solution [17]. The decrease in Cu(II) and Pb(II) percentage removal with the increase of the ions concentration may be due to the restriction of adsorption sites present on the surface of rice straw/Fe_3_O_4_ nanocomposites. At lower initial concentrations, sufficient adsorption sites are available for the sorption of Cu(II) and Pb(II) ions. However, the numbers of Cu(II) and Pb(II) are relatively higher as compared to availability of adsorption sites at higher Cu(II) and Pb(II) concentrations [30]. It can be seen from the Fig. 4b; the percentage removal of Pb(II) is higher than Cu(II) (Fig. 4a). The highest value for Pb(II) than that of Cu(II) may be attributed to the strength of bond formation (stability) along with Jahn-Teller effect that is predominant for Pb(II) complexes [31]. The sorption maxima seems to be inversely proportional to the hydrated ionic radius of the metals, being Pb^2+^ (4.01 Å) > Cu^2+^ (4.19 Å). Hillel [32] explained that the smaller the ionic radius and the greater the valence, the more closely and strongly is the ion adsorbed. Wojnárovits [33] also defined the smaller the hydrated ionic radius, the greater will be the ability to penetrate into smaller pores and therefore greater access to active groups of the adsorbent. On the other hand, the greater the ion’s hydration, the farther it is from the adsorbing surface and the weaker its adsorption [34].

#### Effect of removal time and adsorbent dosage on the removal of Cu(II) and Pb(II)

The Combined influence of removal time and adsorbent dosage was analyzed and it was shown in Fig. 4(c, d). It can be observed as removal time was increased; the removal efficiency of Cu(II) and Pb(II) ions was also increased. The adsorption of ions was fast in the beginning and approached an almost constant value. It implies the achievement of equilibrium. The equilibrium time was obtained at 60 second for both Cu(II) and Pb(II) ions. Complete removal of ions happened in three separate stages as indicated in Fig. 4(c, d). The initial peak portion displayed the high sorption uptake of the Cu(II) and Pb(II) ions on the adsorbent. The second step showed the slow adsorption of ions which illustrated the consumption of all active sites on the surface of adsorbent and reach to saturation or equilibrium stage. The third step showed the equilibrium step in which, the sorption uptake was relatively small [35].

The initial faster rate of metal ion sorption may be attributed to the large number of available sorption sites for adsorption. For the initial blank surface, the adhering possibility is large, and therefore adsorption proceeded with a high rate. The slower adsorption rate at the end is probably due to the saturation of active sites and achievement of equilibrium [36]. As mentioned earlier, by increasing adsorbent dosage the percentage removal of Cu(II) and Pb(II) was increased.

### Optimization and verification of models

The next step was to determine the influences of three independent variables included: initial ion concentration, removal time and adsorbent dosage which shown in Table 4. For this purpose, the response surface methodology, using a central composite rotatable design, was adopted for finding optimal conditions. The Experiment was then conducted under the optimized situations and resulting response was compared to the predicted values. In the numerical optimization, the desired target was chosen for each variable and response from the menu. The possible goals are: maximize, target, minimize, in range, none (only for the response) and set to an exact value (only factors). A minimum and a maximum level must be prepared for each parameter included. Desirability is an objective function that ranges from zero outside of the limits to one at the target [22].

**Table 4:** Predicted and observed response values for validation of models and optimum conditions.

	Independent Variables			Removal of Cu (II) %			Removal of Pb (II) %		
	Initial ions concentration (mg/l)	Removal time(s)	Adsorbent dosage (g)	Exp.	Pre.	RSE(%)	Exp.	Pre.	RSE(%)
**Validation Set**	100	75	0.1	67.55	66.72	1.24	82.65	81.24	1.74
	100	45	0.1	68.12	67.02	1.47	78.95	79.22	0.34
	100	60	0.08	63.32	64.19	1.36	77.83	78.87	1.32
	100	60	0.11	69.46	70.55	1.55	86.21	85.51	0.82
	80	60	0.1	70.18	70.53	0.50	84.81	85.61	0.94
	120	60	0.1	66.70	67.64	1.39	75.21	76.78	2.05
**Optimal conditions**	100	41.96	0.13	75.54	74.033	2.04	-	-	-
	60	59.35	0.13	-	-	-	96.25	94.42	1.94

The optimum parameters for the sorption of Cu(II) and Pb(II) were (100 and 60 mg/L) of initial ion concentration, (41.96 and 59.35 s) of removal time and 0.13 g of adsorbent for both ions, respectively. The experimental results for Cu(II) and Pb(II) gave the reasonable removal ion percentage of 75.54% and 96.25%, respectively. These results confirmed the validity of the models, and the experimental values were determined to be entirely near to the predicted value of 74.033% and 94.42% for Cu(II) and Pb(II), respectively. Percentage of residual standard error (RSE) was then calculated for each response.

Six validation parameters were prepared to confirm the models as shown in Table 4. Experimental values were compared with the predicted values to check the efficiency of the final reduced models. As can be seen the actual values are in agreement with the predicted values, and indicated the models are significant.

### Adsorption mechanism

**Fig 5:**
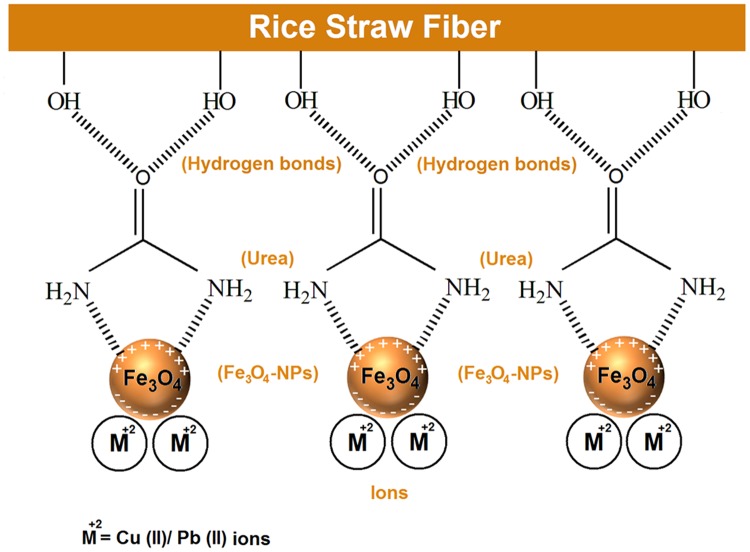
Schematic illustration of adsorption process for Cu(II) and Pb(II) by RS/Fe_3_O_4_-NCs.

With respect to the formation of Fe_3_O_4_ nanoparticles, it can be seen in Fig. 5 which urea was adsorbed on the RS surface through hydrogen bonding between the-OH groups available in RS and the carbonyl group present in the urea. As well as, urea has two NH_2_ groups with negative dipole moments, and the surface of Fe_3_O_4_ nanoparticles has a partial positive charge, hence these different charges, negative and positive, can attract each other [37]. On the other hand, for adsorption of ions by RS/Fe_3_O_4_-NCs when the ions approach to the composite, the Fe_3_O_4_ nanoparticles act as a magnet, and they have been temporary dipole moment and their surface positive charge change to a negative charge, it means there will be cationic interaction between Fe_3_O_4_ nanoparticles and ions. Since there is no new band in the FT-IR results (data not shown), therefore the interaction is physical interaction. In the BET analysis, the surface area and pore volume for RS and RS/Fe_3_O_4_-NCs were obtained 2.45 m^2^ g^−1^, 0.019 cm^3^ g^−1^ and 54.76 m^2^g^−1^, 0.23 cm^3^ g^−1^, respectively. The increasing surface area and pore sizes after coated the nanoparticles and suitable dispersion of nanoparticles as well as superior accessible active sites could be the main reasons for the higher removal of ions by RS/Fe_3_O_4_-NCs in comparison with RS. The better dispersion of nanoparticles on the surface of RS provides more available sites for Cu(II) and Pb(II) to be adsorbed. Beside interaction of NCs with ions, the Fe_3_O_4_ nanoparticles on the surface of RS endowed the adsorbent superparamagnetism to ensure the appropriate magnetic separation after adsorption.

### Biosorption isotherm of Cu(II) and Pb(II)

The equilibrium models are extensively used to determine the amount of the metal ions adsorbed by a specified biomass. The distribution of metal ions between solution and adsorbent is a measure of the equilibrium position and might be explained by one or more isotherms. The models of Langmuir, Freundlich, and Dubinin—Radushkevich (D–R) are some examples and among these most common are the monolayer adsorption developed by Langmuir and the multi-layer adsorption Freundlich models. According to Langmuir, the adsorption occurs at the adsorbent surface in a homogeneous manner and the atoms or ions form a monolayer, do not have mutual interactions, on the adsorbent surface. Although it gives no information about the mechanism, it is used to obtain the uptake capacities of the adsorbents [4]. The adsorption data were obtained by Langmuir equation:

$$\frac{C_{e}}{q_{e}}=\frac{C_{e}}{q_{max}}+\frac{1}{bq_{max}}$$ (6)

where *q*_*e*_ is the amount of metal ion adsorbed at equilibrium (mg/g), *q*_*max*_ is the monolayer sorption capacity (mg/g), *b* is Langmuir constant that is attributed to binding energy of the adsorption system, *C*_*e*_ is the metal ions concentration in the equilibrium.

The separation factor R_L_ is an essential and characteristic index of the Langmuir isotherm model and is often used to judge whether an adsorption process is a thermodynamically favorable process or not: If R_L_> 1, it should be unfavorable adsorption; when R_L_ = 1, it should be a linear adsorption; when 0 < R_L_< 1, it should be a favorable adsorption; when R_L_ = 0, the adsorption is an irreversible [38]. In this study, the R_L_ value was measured to be from 0 to 1 implying that Cu(II) and Pb(II) adsorption by RS/Fe_3_O_4_- NCs was a favorable process [9]. The R_L_ is given by Equation (7). On the contrary, Freundlich model assumes the non-ideal adsorption on heterogeneous surfaces in a multilayer way which given by Equation (8):

$$R_{L}=\frac{1}{1+bC_{0}}$$ (7)

Where *C*_*0*_ is the initial heavy metal ion concentration in (mg/L).

$$lnq_{e}=\;lnK_{f}+\frac{1}{n}lnC_{e}$$ (8)

where *K*_*f*_ and *1/n* are Freundlich constants which are attributed to the adsorption capacity and adsorption intensity, respectively.

**Fig 6:**
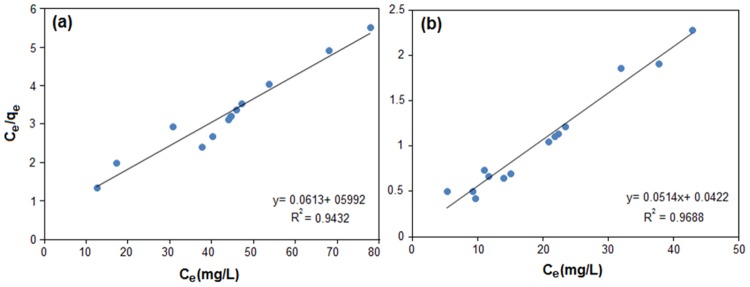
Langmuir isotherm plots of the biosorption of Cu(II) (a), and Pb(II) (b) by RS/Fe_3_O_4_-NCs.

The isotherm parameters of Langmuir and Freundlich were determined from the slope and intercept of linear plots of *C*_*e*_ */q*_*e*_ versus *C*_*e*_ (Fig. 6) and *lnq*_*e*_ versus *lnC*_*e*_, respectively, and the results are shown in Table 5. By comparing the Langmuir isotherm with Freundlich isotherm, the R^2^ values of Freundlich model are less than Langmuir, therefore this model could not explain the connection between the amounts of adsorbed metal ion and their equilibrium concentration in the solutions. Hence, Equilibrium data of the adsorption of Cu(II) and Pb(II) are fitted into the linearized Langmuir isotherm. In addition, the adsorption capacities measured from the Langmuir model were much closer to the experimental quantities of *q*_*e*_ than that of Freundlich model, indicating that Cu(II) and Pb(II) adsorbed by RS/Fe_3_O_4_-NCs formed the monolayer coverage on the adsorbent surface. When the amount of (*n*) from the Freundlich isotherm is higher than 1, it shows the favorable metal ions adsorption on the adsorbent surface. The amount of (*n*) determined in this research is shown in Table 5, demonstrate favorable adsorption of Cu(II) and Pb(II) ions by using RS/Fe_3_O_4_-NCs.

**Table 5:** Isotherm constants for the adsorption of Cu(II), and Pb(II).

	Langmuir isotherm			Freundlich isotherm		
	***q***_***m***_	***b***	***R*** ^***2***^	***K***_***f***_	***n***	***R*** ^***2***^
**Cu (II)**	16.31	1.03	0.9432	1.61	1.85	0.8258
**Pb (II)**	19.45	1.21	0.9688	4.19	1.93	0.826

### Adsorption kinetics model

As mentioned above, the influence of removal time on the adsorption of Cu(II) and Pb(II) ions was significant. The metal ions reached equilibrium at 60 s. The maximum adsorbed was achieved within 30–40 s. It shows that the studied adsorbent has fast kinetics. This may be attributed to the hydrophilic nature of the constituents of RS [14] and the larger surface area of Fe_3_O_4_ nanoparticles [2].

The results obtained were used for two kinetics models including lagergren's pseudo-first order and pseudo-second order kinetics.

The first-order kinetics model is based on the assumption which the adsorption process is controlled by using diffusion stage and the adsorption rate is corresponding to the difference between equilibrium adsorption capacity and adsorbed quantity at time (*t*). So, the adsorption rate can be evaluated by using Equation (9).

**Fig 7:**
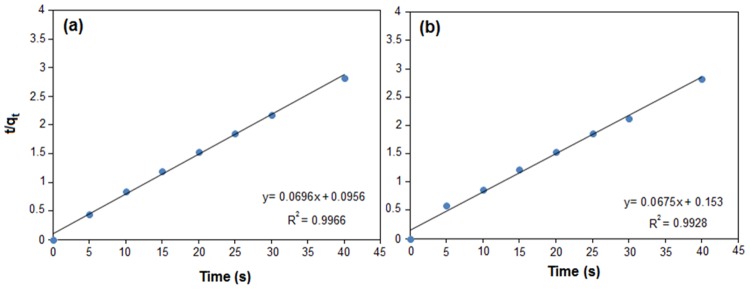
Pseudo- second- order kinetics for (a) Cu(II) and (b) Pb(II).

The pseudo-second order kinetics is also based on the assumption that the adsorption rate is controlled by a chemical adsorption mechanism including sharing or transferring of electron between the adsorbent and adsorbate [39]. So, the adsorption rate can be evaluated using Equation (10), [9]. The initial adsorption rate (*h*_*0*_, mg/g/min) can be evaluated from pseudo-second order kinetic model, as indicated by Equation (11):

$$ln(q_{e}-q_{t})=lnq_{e}-k_{1}t$$ (9)

$$\frac{t}{q_{t}}=\frac{t}{k_{2}q_{e}^{2}}+\frac{t}{q_{e}}$$ (10)

$$h_{0}=k_{2}q_{e}^{2}$$ (11)

where *q*_*e*_ and *q*_*t*_ are the amounts of metal ion adsorbed (mg/g) at equilibrium at time (*t*), respectively. The *k*_*1*_ (min^-1^) parameter is the rate constant of the pseudo-first-order kinetics model, and *k*_*2*_ (g mg^-1^min^-1^) is the rate constant of the pseudo-second-order kinetics model. The kinetics parameters for these two models for various concentrations were determined from the slope and intercepts of the linear plots of *ln (q*_*e*_ *− q*_*t*_) versus (*t*) (result not shown) for pseudo-first-order and *t/q*_*t*_ versus (*t*) (Fig. 7) for pseudo-second-order kinetics, and results are given in Table 6. The validity of the models was obtained by comparing the correlation coefficient (R^2^) of two models. The R^2^ of the pseudo-second-order kinetics model for Cu(II) and Pb (II) was higher than pseudo-first-order kinetics model. Thus, the adsorption results are described by the pseudo-second-order kinetics model. This model provided the best agreement with the experimental results for the Cu(II) and Pb(II) ions adsorption.

**Table 6:** Kinetic parameters for the adsorption of Cu(II) and Pb(II) ions by RS/Fe_3_O_4_-NCs.

	Pseudo- first- order			Pseudo- second- order			
	*K*_*1*_ *(min* ^*-1*^)	*q*_*e*_	*R* ^*2*^	*K*_*2*_ *(g mg* ^*-1*^ *min* ^*-1*^)	*q*_*e*_	*R* ^*2*^	*h*_*0*_
**Cu (II)**	0.0867	5.29	0.9215	0.05	14.36	0.9966	10.31
**Pb (II)**	0.0927	5.59	0.9795	0.03	14.81	0.9928	7.00

The pseudo-second-order kinetics model assumes which the adsorption process occurs on localized sites without interaction between the adsorbates and maximum adsorption corresponds to a saturated monolayer of adsorbates onto the adsorbent surface [40].

### Desorption and reusability

Dilute solution of mineral acids can be used for desorption studies. In this work, HNO_3_ (0.1 M) was chosen as a stripping agent, due to that the metal ions concentration enhanced by increase of H^+^ in water but they have been little changed in 0.1 M acid solution [2].

**Fig 8:**
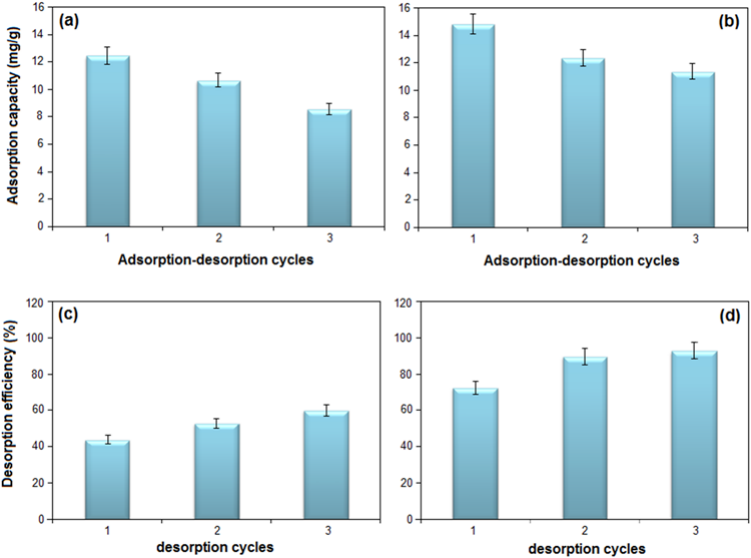
The adsorption-desorption cycles of (a) Cu(II), (b) Pb(II) and desorption efficiency of (c) Cu(II) and (b) Pb(II).

In Fig. 8 the capacity of adsorption of Cu(II) and Pb(II) ions was decreased from 12.47 and 14.86 to 8.56 and 11.38 mg/g for Cu(II) and Pb(II) after three cycles, respectively, and the desorption efficiency of ions was increased from 43.88 and 72.55 to 60.01 and 93.00 wt.% for Cu(II) and Pb(II), respectively. It shows the elution agent is proper to desorb the metal ions from the adsorbent.

The decrease of removal efficiency of Cu(II) and Pb(II) ions could be due to the unavoidable losing weight of adsorbent during desorption process [41].

## Conclusion

This research has explained the probability of response surface methodology (RSM) for investigation the effect of three variable parameters on the Cu(II) and Pb(II) removal from aqueous solution using RS/Fe_3_O_4_-NCs as bioadsorbent. Experiments were carried out based on three independent variables included of initial ion concentration, removal time and adsorbent dosage. The results show RS/Fe_3_O_4_-NCs are good adsorbent for elimination of lead and copper ions and had highly removal efficiency for studying ions. The optimum conditions for the biosorption of Cu(II) and Pb(II) were obtained (100 and 60 mg/L) of initial ion concentration, (41.96 and 59.35 s) of removal time and 0.13 g of adsorbent for both ions, respectively. Equilibrium time was achieved in 60 s for both ions. The isotherm data showed the adsorption of Cu(II) and Pb(II) ions are in agreement with Langmuir model. Kinetics results exhibited the adsorption of ions fitted well with the pseudo-second order kinetic model. The regeneration results confirmed that the prepared nanocomposites can offer excellent reusability from the adsorption medium.
